# A preliminary assessment of spatial variation of water quality of Ratuwa river

**DOI:** 10.1371/journal.pone.0285164

**Published:** 2023-05-02

**Authors:** Arun Kumar Shrestha, Manisha Rai, Jeny Pokhrel, Sailendra Karki, Daya Poudel, Sohan Karki, Swastika Niroula, Ram Prasad Koirala, Ganesh Kumar Shrestha, Buddha Ram Shah

**Affiliations:** 1 Department of Physics, Damak Multiple Campus, Damak, Nepal; 2 Central Department of Physics, Tribhuvan University, Damak, Nepal; 3 M.M.A.M Campus, Tribhuvan University, Biratnagar, Nepal; 4 Pulchowk Campus, Tribhuvan University, Patan, Nepal; 5 Nepal Academy of Science and Technology, Lalitpur, Nepal; University of the Chinese Academy of Sciences, CHINA

## Abstract

This work helps to identify the source of pollution in water and characterize the water quality which is essential to water management for sustainable development. Therefore, the main objective of this work is to evaluate the spatial distribution of the water quality of Ratuwa river and its tributaries. The water samples were collected from six discrete sampling locations and fifteen parameters were tested using respective well-calibrated equipment and standard APHA methods. The physicochemical analysis, water quality index, and correlation matrix method were employed to evaluate the spatial variation of the water quality of Ratuwa river. Turbidity was the most polluting factor in river water. The results showed the spatial variation of the water quality index (WQI) from 39.3 to 70.5, which fell in the range of “good” to “poor” water quality status. None of the water samples was either “excellent” or “unsuitable for drinking.” The water quality was “Poor” upstream and downstream of Ratuwa river due to the high value of turbidity. Chaju river was found to have unpolluted whereas Dipeni river was slightly polluted due to domestic and municipal wastes. Hence, the deterioration of water quality can be attributed to natural and anthropogenic sources.

## Introduction

River water is a major source for providing daily water needs for both animals and plants [1–3] and plays a key role in sustainable development and poverty reduction [4]. In recent decades, rapid population growth, urbanization, and industrialization have significantly deteriorated the water quality of rivers [5, 6] because of their critical role in transporting domestic, municipal, industrial, and agricultural wastes [7]. Such wastes consist of chemicals, pesticides, fertilizers, organic matter, and microorganisms and cause pollution when physicochemical and microbial parameters exceed the desirable limits due to their presence. As a result, it is inappropriate for human consumption, domestic, and industrial applications. The degradation of water quality also reduces the environmental health of river basins and affects the social and economic development of the nation as a result, it is a serious issue throughout the world [8, 9].

Ratuwa is the III category of river in Nepal originating from Siwalik hill. It is fed by precipitation as well as groundwater regeneration and its flow is significantly low during the non-monsoon period. However, about 60–85% of the annual runoff of the river occurs during the monsoon period from July to September. When it emerges out of Chure hill, it passes through Damak Municipality. The damping site of Damak Municipality is also situated on its bank and seepage is highly probable in river water. Damak city also contributes a large volume of domestic, municipal, and chemical wastes. The agricultural area also contributes to some chemical wastes used in the field. An industrial park is also going to build on its bank between Baluwatar and Setumari regions which will also significantly contribute to the chemical and heavy metal wastes in the future. All these scenarios show that the water quality of the Ratuwa river is severely affected due to natural, and anthropogenic activities [10–12]. In this context, it is essential to conduct a comprehensive study to identify the pollution sources and evaluate the water quality for the protection of the aquatic environment. Various methods such as statistical approach, modeling technique, and WQI have been used to evaluate water quality. Among these methods, WQI is the best for river water resource management because it is the simplest and easiest way to analyze the huge data set [13, 14]. Basically, two methods are used for WQI calculation namely Canadian Council of Ministers of the Environment (CCME) WQI and Weighted Arithmetic WQI. CCME WQI requires three statistical parameters (scope, frequency, and amplitude) to calculate the water quality based on the failure of the data to comply with the local quality water legislation [15–17]. However, weighted arithmetic WQI deals predominantly with selected physicochemical parameters, assignment of weightage, and aggregation by a multiplicative form. It summarizes the combined influence of several environmental parameters and provides a single efficient value that reflects the overall water quality [18, 19]. For example, an evaluation of WQI of the spatial distribution of the Sungai Setiu basin in Malaysia showed that water quality was almost constant over the past 10 years [20].

In fact, limited works are conducted to evaluate the water quality in various rivers of Nepal either a using statistical approach or modeling technique [21, 22]. The author’s team conducted preliminary works at a specific time and location of Ratuwa river to predict the suitability of water [23, 24] using WQI. However, it was not sufficient to represent the spatial variation of water quality along the river due to the heterogeneous nature of pollutants [25]. The present study was undertaken to access the spatial variation of water quality along the river from the Chapeti (upstream) to Setumari (downstream) in terms of physicochemical parameters from six sampling locations. The assessment of water quality and probable pollution sources were also explored using some statistical tools. This is the first study representing a detailed assessment of the pollution status of the whole stretch of Ratuwa river in Damak and provides the baseline information for the water management system and conversation strategies for the Ratuwa river and its tributaries to improve the water quality and its environment [26].

## Materials and methods

### Study area

**Fig 1:**
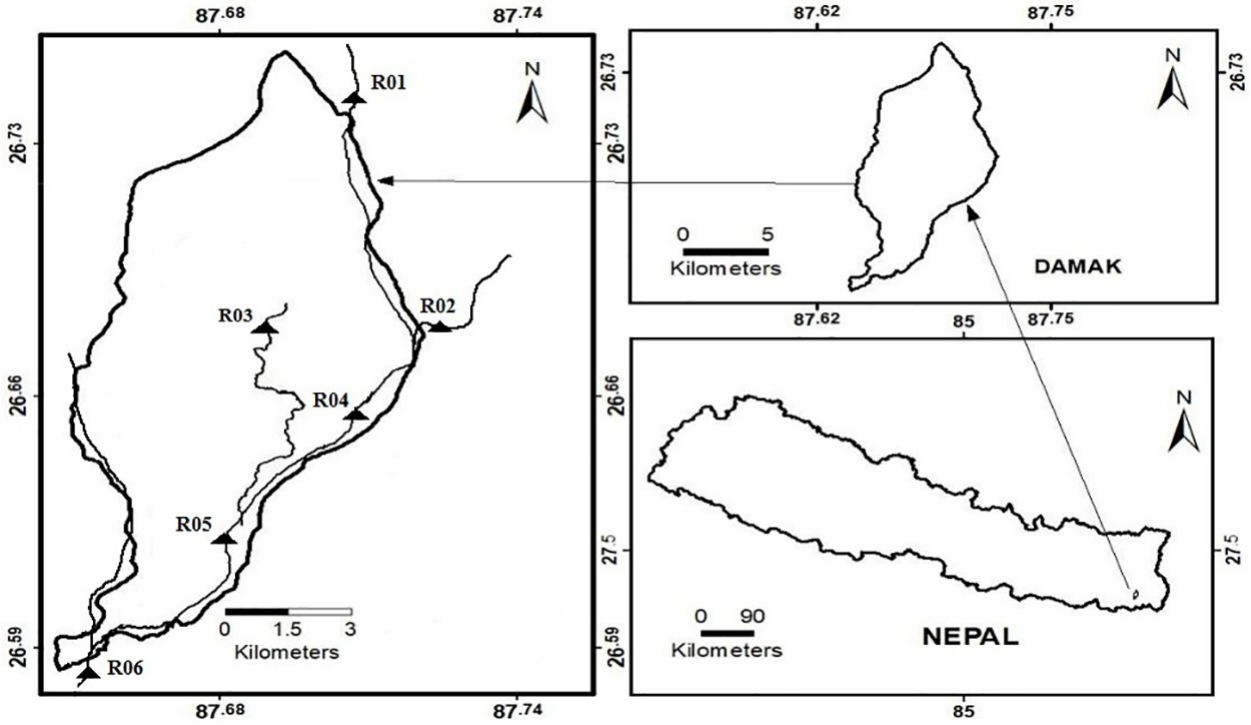
Map of the study area with sampling stations.

The Ratuwa river is a small perennial river in the eastern part of Nepal. It originates from Siwalik hill and empties into the Kankai river near Bihar in India. It has three major tributaries when it comes out from Chure hill mainly: Chaju, Dipeni, and Mawa river. Mawa river is almost empty in the dry season so it is not considered in our study. The first sampling station was Chapeti (26^*o*^ 44′ 20.4″ N and 87^*o*^ 42′ 47.2″ E). It was chosen at the point where mainstream had just come out from Chure hill. In order to represent the water quality of Chaju river, the sampling station was fixed at Tukure (26^*o*^ 40′ 45.3″ N and 87^*o*^ 43′ 7.3″ E). There is a small forest and agricultural land around it with no human settlement and almost free from anthropogenic activities. Hariom Colony (26^*o*^ 40′ 1.4″ N and 87^*o*^ 41′ 35.9″ E) was also fixed to represent the water quality of the Dipeni River due to anthropogenic activities. The Dipeni river always collects domestic and municipal wastes because there is a dense human settlement around it. Another sampling station was chosen at Ratuwa Bridge (26^*o*^ 39′ 12.6″ N and 87^*o*^ 42′ 13.4″ E) at the mainstream. At this spot, some domestic waste was accumulated through the drain. Baluwatar (26^*o*^ 37′ 31.5″ N and 87^*o*^ 40′ 41.6″ E) is located in the mainstream where Dipeni river mixed with Ratuwa river and it is choosen to evaluate the change in water quality of the mainstream due to Dipeni river. The last station is Setumari (26^*o*^ 45′ 6.6″ N and 87^*o*^ 38′ 45.4″ E), downstream of the Damak where Mawa river comes to mix with Ratuwa river. This point is chosen to find the overall water quality of Ratuwa river due to human activity in Damak city. All six sampling locations, including their tributaries, are shown in Fig 1. Only Damak section of Ratuwa is the main focus of the study. It has a subtropical monsoon, with summer temperatures ranging from 32 to 35°C, winter temperatures ranging from 8 to 15°C, and an average annual rainfall of 2448 mm [27].

### Sample analysis

The samples were collected from all sites on 17^th^ March 2019 in clean one-liter plastic bottles. Sample bottles were first rinsed with sample water, then sealed after sampling, and labeled properly on site. The samples were collected by pumping from 5 to10 cm below the surface water to avoid contamination of the river basin [28]. The instrument and method used to determine fifteen physicochemical parameters are reported in Table 1. The quality control of laboratory equipment is according to ISO 17025:2017 documentation process and the detection limit for the instrument used for chemical analysis is 0.05mg/L.

**Table 1:** The instruments and methods used for the analysis of water parameters with relative weight calculation.

S.N	Parameters	Instrument used	Methods adopted	Standard values (S_i_)	Recommended Agency	Assigned weight (W_a_)	Relative weight (W_r_)
1.	Temperature	Thermometer	-	-	-	-	-
2.	pH	PH meter (HI98107)	APHA– 4500H+	7.5	NDWQS	4	0.100
3.	EC	Conductivity meter (model PCD-431)	APHA– 2510	300	WHO	5	0.125
4.	TDS	Conductivity meter (model PCD-431)	APHA– 2540C	500	WHO	4	0.100
5.	DO	DO meter (model DO-5509)	APHA– 5520B	6	NDWQS	5	0.125
6.	Turbidity	Turbidity meter(EI Model 331)	APHA– 2130B	5	NDWQS	4	0.100
7.	Nitrate as NO_3_-N	UV-VIS Spectrophotometer	APHA– 4500	-	-	-	-
8.	Chloride as Cl	Titrimetric	APHA– 2500B	250	NDWQS	3	0.075
9.	TH as CaCO_3_	Titrimetric	APHA– 2340	500	NDWQS	2	0.050
10.	TA as CaCO_3_	Titrimetric	APHA– 2320	200	WHO	2	0.050
11.	Sulphate	Gravimetric	APHA– 4500	250	NDWQS	4	0.100
12.	Sodium	Atomic Absorption Spectrometer	APHA– 3500	200	WHO	2	0.050
13.	Potassium	Atomic Absorption Spectrometer	APHA– 3500	12	NDWQS	2	0.050
14.	Calcium	Titrimetric	APHA– 3500	75	WHO	2	0.050
15.	Magnesium	Titrimetric	APHA– 3500	50	WHO	1	0.025
16.						W_a_ = 40	

The digital turbidity meter of EI model 331 was used to measure turbidity that has a resolution of 1NTU and ranged from 0–1000 NTU with an accuracy of ±3% FS, ±1 digit. The photodiode is used to detect the suspension in the solution and amplified to display on a 3A½ digit seven segments LED display.

The conductivity meter of model PCD-431 is a portable device that can operate up to 50°C and humidity less than 80%. The instrument’s resolution is 1μS/cm while measuring the electrical conductivity and 1ppm while measuring the TDS and the accuracy is ± (3% F.S. +1digit). The pH tester (HI98107) is a portable instrument that has a resolution of 0.1pH and accuracy ± 0.1pH. It can operate within a temperature range of 0–50°C and in a 100% humid environment.

Thermo Scientific^™^ GENESYS 10 UV UV-Vis spectrophotometer was utilized for nonmetal analysis (Nitrate). It has a high-intensity xenon lamp to perform accurate analysis over the entire wavelength range of 190-1100nm with scan speeds up to 3600 nm/minute and offers an excellent value providing high performance, reliability, and ease of use.

The iCE 3000 Series Atomic Absorption Spectrometer was used to estimate the concentration of Na^+^ and K^+^ in flame emission mode. It is based on the principle of absorption of light in the ground state of their ions. Due to their volatile nature, it measures elements up to the trace (μg/mL) and ultra-trace (sub-μg/mL) levels with great accuracy and acceptable precision. Analytical performance was controlled by Thermo Scientific SOLAAR software under a Windows^®^ operating system.

### Water quality index

To calculate the WQI, each measure was given an assigned weight (W_a_) from 1 to 5 based on its relative impact on total water quality [29, 30]. The parameters with the greatest impact on water quality, such as EC and DO, were given a weight of 5, while the least impact parameter, such as magnesium was given a weight of 1. The relative weight for each parameter was then determined using Eq (1) in Table 1.

$$W_{r}=\;\frac{{\left(W_{a}\right)}_{i}}{\sum_{i=1}^{n}\left(W_{a}\right)i}$$ (1)

where, n represents the number of parameters taken into account for WQI.

Then, using Eq (2), the quality rating scale Q_i_ was determined for each of the i^th^ parameters.

$$Q_{i}=\;\left(\frac{C_{i}-V_{i}}{S_{i}-V_{i}}\right)\times 100$$ (2)

Where Q_i_ refers to quality rating and C_i_, S_i_, and V_i_ are the measured concentration, standard reference, and ideal values for each parameter respectively. The standard reference values were employed by Nepal Drinking Water Quality Standards (NDWQS) and WHO [31, 32]. The ideal value of pH = 7.0 and DO = 14.6 was chosen, whereas, for other parameters, it was set to zero [33]. The sub-indices were used to calculate WQI, which was obtained using Eq (3). Finally, WQI is determined by Eq (4)

$$SI_{i}=\;W_{r}\;\times \;Q_{i}$$ (3)

$$WQI=\;\overset{n}{\underset{i=1}{\sum }}{\left(SI\right)}_{i}$$ (4)

The WQI and water quality status were classified using the proposed scaling by Nayak and Mohanty 2018 [34]. In this scale, a low score represents a good water quality status. According to the WQI values, water quality status was divided into five degrees: “excellent” (0–25), “good” (26–50), “Poor” (51–75), “very poor” (76–100), and “unsuitable for drinking purposes” above 100.

## Results and discussion

Descriptive statistics of the analyzed elements have been reported in Table 2 including the number of observations, means, minima, maxima, and standard deviations. The results of measured parameters were compared with the standard values established by WHO and NDWQS guidelines.

**Table 2:** Descriptive statistics of water quality parameters of Ratuwa river and its tributaries.

S.N	Parameters	Unit	No of Obs.	Minimum	Maximum	Mean	Standard Deviation
1	Temperature	°C	6	23.5	38.7	29.05	5.17
2	PH	**-**	6	6.5	7.9	7.40	0.50
3	EC	μS/cm	6	137	285	239	54.20
4	TDS	mg/L	6	91	189	159.17	36.02
5	DO	mg/L	6	3.3	6.5	5.42	1.20
6	Turbidity	NTU	6	2.0	11	5.67	3.79
7	Nitrate	mg/L	6	<0.05	<0.05	—	—
8	Chloride	mg/L	6	1	8	4.17	2.79
9	TH	mg/L	6	29	84	69.67	20.81
10	TA	mg/L	6	47.6	103.70	85.28	20.07
11	Sulphate	mg/L	6	3	44	21.50	13.37
12	Sodium	mg/L	6	6.65	9.64	7.78	1.19
13	Potassium	mg/L	6	4.84	5.98	5.27	0.43
14	Calcium	mg/L	6	8.02	30.86	21.38	7.83
15	Magnesium	mg/L	6	0.73	7.05	4.17	2.31

### pH

pH shows the acidic or basic nature of water. The mild alkaline nature of water at all sites except the slightly acidic water of Hariom Colony suggests there may be the presence of minerals in the water. The low value of pH at the site of Hariom Colony is due to the influence of the drainage water of Damak Municipality, which contains a significant decay of organic matter with increasing organic acids [35]. Overall, all the values were within the desirable limit of 6.5–8.5 given by NDWQS.

### EC

It is another water quality parameter that measures the ionic condition of water and is affected by temperature, the concentration of impurities, and the mobility of ions [36]. The lowest value of EC was 137μS/cm in Tukure and the highest value was 285μS/cm in Setumari. The moderate value of EC in water may be due to domestic or agricultural runoff and calcium carbonate weathering [37]. A low value of EC at Tukure indicates that there is no contamination of water from natural and anthropogenic activities. It is also observed that all values are within the recommended value of 300μS/cm prescribed by NDWQS. However, a nominal increase of EC downstream of water indicates the gradual contamination in river water.

### TDS

It represents the total amount of dissolved organic and inorganic salts in water. The common inorganic salts contain calcium, magnesium, sodium, and potassium ions as well as bicarbonate, carbonate, chloride, and sulphate ions. The minimum concentration of TDS was 91mg/L at Tukure and the maximum concentration was observed at Setumari with 189 mg/L followed by 177 mg/L at Hariom Colony and Baluwatar, 173 mg/L at Chapeti, and 148 mg/L at Ratuwa Bridge. However, there is no significant spatial variation in TDS except at Tukure and it is well under the WHO-recommended limit of 500 mg/L. The TDS levels in water may rise as a result of natural and anthropogenic activities, altering the taste of foods and beverages and making their consumption less desirable [38].

### DO

It is an important factor in determining water quality, ecological state, and overall water health and measures the dissolved oxygen in water [39]. DO should be larger than 4 mg/L for aquatic creature viability and 6 mg/L for drinking reasons [40]. The DO levels in the study area ranged from 3.3 to 6.5 mg/L. However, three sampling sites namely: Chapeti, Ratuwa Bridge, and Setumari have DO values in a permissible range for drinking purposes and represent the better quality of water for aquatic organism survival and growth. The lowest value of DO at Hariom Colony is due to the presence of domestic and municipal wastes and at Tukure, it is due to the high surface temperature of the water.

### Turbidity

Turbidity is a measure of how cloudy water is, making it impossible to see through it. It is due to suspended or colloidal particles, as well as chemical precipitation. For drinking purposes, the NDWQS recommends a turbidity limit of 5 NTU. In this study, turbidity values ranged from 2 NTU to 11 NTU. In two sampling stations, Chapeti and Setumari, turbidity was 11 and 9.80 NTU, respectively. It is due to the soil runoff and high deposition of sediments in the river. In such turbid water, it may also contain disease-causing microorganisms [41].

### Chloride

Chloride is a naturally occurring ion that can be found in a variety of rocks and soils. It is a dominating anion in surface water. However, excessive consumption of chloride ions over a long period of time poses health risks [42]. The chloride concentrations were exceptionally low when compared to the recommended value of 250 mg/L set by the NDWQS and WHO and its concentration in the sampled water ranged from 1 to 8 mg/L. Anthropogenic activities and saline residue leaching may be responsible for these chloride ion levels in water [43].

### Total hardness

It is a measure of mineral content in water, particularly calcium and magnesium. “Water with a hardness of less than 60 mg/L is called soft, 60–120 mg/L moderately hard, 120–180 mg/L hard, and more than 180 mg/L very hard” [44]. The minimum concentration of TH was 29 mg/L at Tukure and the maximum concentration was 84 mg/L at Baluwatar. All samples are moderately hard except water at Tukure. Since Ratuwa and Chaju river originates from Siwalik hill and water may interact with limestone and sedimentary rock and calcium-bearing minerals in its course. However, the Chaju river may transport little calcium-bearing mineral water on its course as a result, it has soft water at Tukure (29mg/L). Hard water doesn’t have a serious health impact but is not forever good for bathing, laundering, and washing, however, its lower value is excellent in preventing pipe corrosion [45].

### Total alkalinity

The ability of water to counteract acidity is referred to as alkalinity. The sources of TA in rocks and soil are hydroxide, carbonates, and bicarbonates. It varied from 47.6 mg/L to 103.70 mg/L but remained within the WHO’s recommended limit of 200 mg/L. The sampling station at Tukure had the lowest concentration and the sampling station at Setumari had the highest concentration. A spatial variation of TA among these stations indicates that there is little influence of anthropogenic activities.

### Sulphate

It is naturally present in water due to the oxidation of sulphate ores. In this study, the sulphate concentration varied from 3–44 mg/L which is extremely low compared to the standard value of 250mg/L prescribed by NDWQS for drinking purposes. The abundant concentration of sulphate may cause gastrointestinal problems [46].

### Sodium

In water, sodium can occur naturally in rocks and soil. Our body needs it to maintain normal blood pressure levels, normal nerve, and muscle functions [47]. The lowest sodium level was 6.65 mg/L in Ratuwa river and the highest level was 9.64 mg/L in Hariom Colony. The concentration of sodium at all stations did not pose any water quality problem because it was still below the recommended value of 200 mg/L for drinking purposes.

### Potassium

Potassium can be found in abundance in igneous and sedimentary rocks. Potassium minerals provide resistance to weathering and disintegration, therefore its concentration in natural water is usually very low. Potassium is an essential component of plant growth. In the study area, potassium concentration ranged from 4.84 mg/L to 5.98 mg/L.

### Calcium

The major cations in river water are calcium and magnesium, which are mostly responsible for water hardness. Calcium concentrations in this study ranged from 8.02 to 30.86 mg/L, while magnesium concentrations ranged from 0.73 to 7.05 mg/L. Both calcium and magnesium concentrations were below the WHO recommended values of 75 mg/L and 50 mg/L for drinking purposes, respectively. Calcium is obtained by dissolving carbonate minerals (e.g., calcite, dolomite, and aragonite). Similarly, magnesium, which is always found in low concentrations in all types of water, comes from Mg-bearing minerals and magnesium sulphate minerals [48].

The principal ion chemistry revealed that calcium was the most abundant cation, with Ca^2+^ > Na^+^ > Mg^2+^ > K^+^ recorded in Chapeti, Hariom Colony, and Ratuwa bridge and Ca^2+^ > Na^+^ > K^+^ > Mg^2+^ in Tukure, Baluwatar, and Setumari. The breakdown of organic materials and fertilizers produces potassium ions in water [49, 50] as a result, K^+^ is sometimes higher than Mg^2+^, and vice versa. The high level of potassium in river water indicates that agricultural products and fertilizers have been used extensively in the soil. Overall, alkali earth elements (Ca^++^ + Mg^++^) outnumber alkaline earth elements (Na^+^ + K^+^). Again, the typical molar ratios of Na+Cl−=2.88 and K+Cl−=1.15 in the Ratuwa river are substantially greater than the comparable ratios in marine water, which are Na+Cl−=0.86 and K+Cl−=0.02. This demonstrates that atmospheric contributions have a negligible impact on river water and decrease as the distance increases from the sea [51].

### Water quality index

It was calculated from thirteen parameters using the weighted arithmetic method and tabulated in Table 3 to show the spatial distribution of water quality. In all sampling stations, WQI values have more than 50 and represent poor water quality except at Tukure station where it is only 39.3 and represents good water quality. However, it exhibits a low value of DO (4.9 mg/L) at that station which is likely owing to the high temperature, as higher temperature reduces the solubility of oxygen in water [52]. In upstream and downstream of the river basin, especially at Chapeti and Setumari, the WQI is relatively higher as compared to other stations. In Chapeti station, it is due to the soil runoff and high deposition of sediments in the river. In Setumari station, it is due to the deposition of sediments and other impurities by anthropogenic activities in the water. A low value of DO and moderate value of EC at Hariom Colony clearly indicate that Dipeni river is comparatively more polluted by domestic and municipal wastes. The Ratuwa Bridge and Baluwatar station have WQI values of 53.5 and 52.1, respectively and it indicates that these stations are also slightly polluted by anthropogenic activities. Moreover, a higher value of calcium increases the hardness of water and hence the conductivity. Many natural and anthropogenic factors, such as soil type, wastewater, and fertilization, may be contributing to the decline of water quality at these sites. In this study, water samples were collected in only one from each station which was limited. Seasonal sampling from each station is needed for better understanding and distinguishing the status of water quality [53]. Some parameters such as ammonium nitrogen, nitrate, nitrite, phosphorus, and BOD were important to evaluate pollution due to anthropogenic activities. However, they were not analyzed in this study. A statistical approach such as principal component analysis (PCA) could be applied to identify the sources of pollution in river water and it is also missing in this study. Therefore, this study only presents a preliminary result and more accurate assessments of water quality should be carried out.

**Table 3:** Spatial distribution of water quality index.

Sampling location	WQI values	Water quality rating
Chapeti (R01)	70.5	Poor
Tukure (R02)	39.3	Good
Hariom Colony (R03)	57.0	Poor
Ratuwa Bridge (R04)	53.5	poor
Baluwatar (R05)	52.1	Poor
Setumari (R06)	69.4	Poor

The WQI of the present study was compared with the I category (Marshyangdi river) and II category (Bagmati river) of rivers in Nepal. The analyzed parameters and WQI values are tabulated in Table 4. In the Marshyangdi river, BOD, ammonium nitrogen, and phosphate were also measured, and in the Bagmati river, heavy metal ions such as chromium, lead, and TSS were also measured. The WQI values of Ratuwa, Bagmati, and Marshyangdi rivers are 57.1, 37.1, and 39.3, respectively. The Weighted Arithmetic Mean method was used to evaluate the WQI in Ratuwa and Marshyangdi rivers and CCME WQI method was used in Bagmati river. WQI and quality rating depend upon the method deployed to evaluate it. Even if, the same method is applied, even more, it depends upon which parameters are analyzed and how the relative weight is calculated. In the present study, relative weight (*W*_*r*_) is calculated by assigning the number from 1 to 5 depending upon the weight of each parameter. But in the Marshyangdi river, relative weight (*W*_*r*_) was calculated by assigning each parameter by 1. As a result, the WQI value of the Marshyangdi river is slightly less than that of the Ratuwa river.

**Table 4:** Comparison of WQI of present study with literatures.

SN	Name of river	Measured parameters	WQI	Evaluation method	references
1	Ratuwa	pH, EC, TDS, DO, Turbidity, TH, TA *Cl*^−^, $SO_{4}^{--}$, *Na*^+^, *K*^+^, C*a*^++^, *Mg*^++^	57.1	Arithmetic Weighted	Present study
2	Bagmati	pH, DO, BOD, TSS, TDS, *Cl*^−^, $NO_{3}^{-}$, *Cr*^+++^, *Pb*^++^, $PO_{4}^{--}$	37.1	CCME WQI	Regmi et al. 2017 [54]
3	Marshyangdi	pH, DO, BOD, TDS, NH_3_, *Cl*^−^, $NO_{3}^{-}$, $PO_{4}^{--}$	39.3	Arithmetic Weighted	Singh et al. 2021 [55]

### Correlation matrix analysis

Pearson’s correlation matrix was built to gain an idea of the linear relationship between WQI and water quality measures. The correlation coefficient has a value between +1 to -1, where ±1 refers to a perfect linear relationship between the two variables and 0 refers to a nonlinear relationship between two variables. Table 5 shows the correlation matrix of different parameters with WQI. EC, TA, and TDS show a moderate correlation with WQI. The other parameters have a weakly positive correlation with the WQI, except for chloride and potassium, as they have a negative correlation with the WQI. The pH exhibited a highly positive correlation with DO (*r* = 0.989) as the reaction of hydrogen ions and oxygen with water shifted according to the pH value. Another pretty strong correlation of EC was detected with TDS (*r* = 0.999), TH (*r* = 0.914), and TA (*r* = 0.989) as the conductivity variation indicates the status of inorganic contamination. Calcium has a negative correlation with sodium (*r* = −0.115) and potassium (*r* = −0.035). It is due to the cation exchange process; sodium and potassium ions replace calcium ions along with river water flow [56]. The presence of cations and anions from the same calcium and magnesium compounds results in a significant correlation of TH with TA (*r* = 0.914) in river water [57]. Even TA is also more than TH indicating the ions exchanging process in the carbonated hardness [58]. The ion exchange process in water is a natural water-softening process in which sodium ions replace calcium and magnesium ions. The concentration of sodium increases in the given order of sampling sites: Ratuwa river < Tukure < Setumari < Baluwatar < Chapeti < Hariom, whereas the concentration of chloride does not follow this pattern. Again, sodium and chloride ions have no substantial correlation. So, it is concluded that they are derived from different sources rather than their combined forms.

**Table 5:** Pearson’s correlation matrix of water quality parameters.

	pH	EC	TDS	DO	Turbidity	*Cl* ^−^	TH	TA	$SO_{4}^{--}$	*Na* ^+^	*K* ^+^	*Ca* ^++^	*Mg* ^++^	WQI
pH	1													
EC	0.038	1												
TDS	0.043	**0.999** *	1											
DO	**0.989** *	0.071	0.074	1										
Turbidity	0.185	0.406	0.394	0.317	1									
*Cl* ^−^	-0.346	-0.081	-0.070	-0.472	**-0.880** **	1								
TH	**0.372**	**0.914** *	**0.918** *	0.385	0.307	-0.044	1							
TA	0.102	**0.989** *	**0.988** *	0.152	0.505	-0.214	**0.914** *	1						
$SO_{4}^{--}$	0.595	0.271	0.263	0.623	0.590	-0.599	0.285	0.315	1					
*Na* ^+^	-0.653	0.428	0.425	-0.578	0.284	0.038	0.231	0.389	-0.503	1				
*K* ^+^	-0.133	-0.105	-0.105	-0.228	-0.328	0.531	-0.145	-0.222	0.175	-0.235	1			
*Ca* ^++^	0.475	**0.845** **	**0.847** **	0.471	0.294	-0.137	**0.892** **	**0.845** **	0.581	-0.115	-0.035	1		
*Mg* ^++^	-0.055	0.226	0.235	-0.030	-0.032	0.217	0.376	0.236	-0.612	0.641	-0.350	-0.072	1	
WQI	0.269	**0.818** **	**0.813** **	0.369	0.804	-0.618	0.774	**0.862** **	0.471	0.359	-0.455	0.712	0.197	1

* represents highly correlation with a significant level at 0.01

** represents moderately correlation with a significant level at 0.05

## Conclusion

In this study, the spatial variation of water quality of Ratuwa river and its tributaries was evaluated using physicochemical analysis, water quality index, and correlation matrix method. There is no exceptionally low and high value in any parameters and WQI and demonstrates no significant change in water quality at different stations. The WQI shows that the quality rating is “Good” in Tukure and “Poor” in all sampling stations. None of the water samples was either “excellent” or “unsuitable for drinking”. Chaju river is less polluted whereas Dipeni river at Hariom colony is more polluted from anthropogenic activities. A high value of WQI at Chapeti and Setumari is due to the soil runoff and high deposition of sediments in the river. Ratuwa Bridge and Baluwatar stations are also slightly affected by anthropogenic activities. Moreover, a correlation matrix shows that EC, TA, and TDS have a high degree of positive correlation with WQI. Moreover, the knowledge gained from this study can be used in the water quality management of water bodies and to identify the major sources of pollution in subsequent research.

## Supporting information

S1 Table Physicochemical parameters of different sampling points. (DOCX)

S2 Table Calculation of WQI. (DOCX)
