# Supplementary material for: A preliminary assessment of spatial variation of water quality of Ratuwa river
Source: PLoS One. 2023 May 2;18(5):e0285164. doi: 10.1371/journal.pone.0285164 (PMC10153709; doi:10.1371/journal.pone.0285164)
Supplement: S1 Table — (DOCX) [file pone.0285164.s001.docx]

S1 Table. Physicochemical parameters of different sampling points

| **S.N** | **Parameters** | **Unit** | **Sample Sites** | | | | | |
| --- | --- | --- | --- | --- | --- | --- | --- | --- |
|  |  |  | **R01** | **RO2** | **RO3** | **RO4** | **RO5** | **RO6** |
| 1 | Temperature | ^0^C | 28.0 | 38.7 | 23.5 | 26.1 | 28.8 | 29.2 |
| 2 | PH | **-** | **7.7** | 7.2 | 6.5 | 7.9 | 7.5 | 7.6 |
| 3 | EC | µS/cm | 260 | 137 | 266 | 221 | 265 | 285 |
| 4 | TDS | Ppm | 173 | 91 | 177 | 148 | 177 | 189 |
|  | DO | mg/L | 6.4 | 4.9 | 3.3 | 6.5 | 5.4 | 6.0 |
|  | Turbidity | NTU | 11 | 4.10 | 4.40 | 2 | 2.70 | 9.80 |
|  | Nitrate | mg/L | <0.05 | <0.05 | <0.05 | <0.05 | <0.05 | <0.05 |
|  | Chloride | mg/L | 1 | 4 | 6 | 5 | 8 | 1 |
|  | TH | mg/L | 83 | 29 | 68 | 74 | 84 | 80 |
|  | TA | mg/L | 96.90 | 47.6 | 93.50 | 79.90 | 90.10 | 103.70 |
|  | Sulphate | mg/L | 24 | 19 | 3 | 16 | 23 | 44 |
|  | Sodium | mg/L | 8.81 | 6.95 | 9.64 | 6.65 | 7.53 | 7.07 |
|  | Potassium | mg/L | 4.91 | 5.52 | 5.09 | 4.84 | 5.98 | 5.26 |
|  | Calcium | mg/L | 21.64 | 8.02 | 18.04 | 23.65 | 26.05 | 30.86 |
|  | Magnesium | mg/L | 7.05 | 2.19 | 5.59 | 4.86 | 4.62 | 0.73 |
