# Supplementary material for: A preliminary assessment of spatial variation of water quality of Ratuwa river
Source: PLoS One. 2023 May 2;18(5):e0285164. doi: 10.1371/journal.pone.0285164 (PMC10153709; doi:10.1371/journal.pone.0285164)
Supplement: S2 Table — (DOCX) [file pone.0285164.s002.docx]

Table S2 Calculation of WQI at Chapeti (R01)

| **S.N** | **Parameters** | **Observed values (C_i_)** | **Standard values (S_i_)** | **Relative weight (W_r_)** | **Quality rating (Q_i_)** | **Sub-indices (SI) values** |
| --- | --- | --- | --- | --- | --- | --- |
|  | PH | **7.7** | 7.5 | 0.100 | 140 | 14.00 |
|  | EC | 260 | 300 | 0.125 | 86.667 | 10.83 |
|  | TDS | 173 | 500 | 0.100 | 34.6 | 3.46 |
|  | DO | 6.4 | 6 | 0.125 | 95.349 | 11.92 |
|  | Turbidity | 11 | 5 | 0.100 | 220 | 22.00 |
|  | Chloride | 1 | 250 | 0.075 | 0.4 | 0.03 |
|  | TH | 83 | 500 | 0.050 | 16.6 | 0.83 |
|  | TA | 96.90 | 200 | 0.050 | 48.45 | 2.42 |
|  | Sulphate | 24 | 250 | 0.100 | 9.6 | 0.93 |
|  | Sodium | 8.18 | 200 | 0.050 | 4.09 | 0.20 |
|  | Potassium | 4.91 | 12 | 0.050 | 40.917 | 2.04 |
|  | Calcium | 21.64 | 75 | 0.050 | 28.853 | 1.44 |
|  | Magnesium | 7.05 | 50 | 0.025 | 14.1 | 0.35 |
|  |  |  |  |  | WQI = ∑SI = 70.5 | |

Table S3 Calculation of WQI at Tukure (R02)

| **S.N** | **Parameters** | **Observed values (C_i_)** | **Standard values (S_i_)** | **Relative weight (W_r_)** | **Quality rating (Q_i_)** | **Sub-indices (SI) values** |
| --- | --- | --- | --- | --- | --- | --- |
|  | PH | 7.2 | 7.5 | 0.100 | 40 | 4.0 |
|  | EC | 137 | 300 | 0.125 | 45.67 | 5.71 |
|  | TDS | 91 | 500 | 0.100 | 18.2 | 1.82 |
|  | DO | 4.9 | 6 | 0.125 | 112.79 | 14.10 |
|  | Turbidity | 4.10 | 5 | 0.100 | 82 | 8.20 |
|  | Chloride | 4 | 250 | 0.075 | 1.6 | 0.12 |
|  | TH | 29 | 500 | 0.050 | 5.8 | 0.29 |
|  | TA | 47.6 | 200 | 0.050 | 23.8 | 1.19 |
|  | Sulphate | 19 | 250 | 0.100 | 7.6 | 0.76 |
|  | Sodium | 6.95 | 200 | 0.050 | 3.475 | 0.17 |
|  | Potassium | 5.52 | 12 | 0.050 | 46 | 2.30 |
|  | Calcium | 8.02 | 75 | 0.050 | 10.693 | 0.53 |
|  | Magnesium | 2.19 | 50 | 0.025 | 4.38 | 0.11 |
|  |  |  |  |  | WQI = ∑SI = 39.3 | |

Table S4 Calculation of WQI at Hariom Colony (RO3)

| **S.N** | **Parameters** | **Observed values (C_i_)** | **Standard values (S_i_)** | **Relative weight (W_r_)** | **Quality rating (Q_i_)** | **Sub-indices (SI) values** |
| --- | --- | --- | --- | --- | --- | --- |
|  | PH | 6.5 | 7.5 | 0.100 | 100 | 10.0 |
|  | EC | 266 | 300 | 0.125 | 88.667 | 11.1 |
|  | TDS | 177 | 500 | 0.100 | 35.4 | 3.54 |
|  | DO | 3.3 | 6 | 0.125 | 131.395 | 16.42 |
|  | Turbidity | 4.40 | 5 | 0.100 | 88 | 8.80 |
|  | Chloride | 6 | 250 | 0.075 | 2.4 | 0.18 |
|  | TH | 68 | 500 | 0.050 | 13.6 | 0.68 |
|  | TA | 93.5 | 200 | 0.050 | 46.75 | 2.33 |
|  | Sulphate | 3 | 250 | 0.100 | 1.2 | 0.12 |
|  | Sodium | 9.64 | 200 | 0.050 | 4.82 | 0.24 |
|  | Potassium | 5.09 | 12 | 0.050 | 42.417 | 2.12 |
|  | Calcium | 18.04 | 75 | 0.050 | 24.053 | 1.20 |
|  | Magnesium | 5.59 | 50 | 0.025 | 11.18 | 0.28 |
|  |  |  |  |  | WQI = ∑SI = 57.0 | |

Table S5 Calculation of WQI at Ratuwa Bridge (RO4)

| **S.N** | **Parameters** | **Observed values (C_i_)** | **Standard values (S_i_)** | **Relative weight (W_r_)** | **Quality rating (Q_i_)** | **Sub-indices (SI) values** |
| --- | --- | --- | --- | --- | --- | --- |
|  | PH | 7.9 | 7.5 | 0.100 | 180 | 18.0 |
|  | EC | 221 | 300 | 0.125 | 73.667 | 9.21 |
|  | TDS | 148 | 500 | 0.100 | 29.6 | 2.96 |
|  | DO | 6.5 | 6 | 0.125 | 94.186 | 11.77 |
|  | Turbidity | 2 | 5 | 0.100 | 40 | 4.0 |
|  | Chloride | 5 | 250 | 0.075 | 2 | 0.15 |
|  | TH | 74 | 500 | 0.050 | 14.8 | 0.74 |
|  | TA | 79.90 | 200 | 0.050 | 39.95 | 2.00 |
|  | Sulphate | 16 | 250 | 0.100 | 6.4 | 0.64 |
|  | Sodium | 6.65 | 200 | 0.050 | 3.325 | 0.17 |
|  | Potassium | 4.84 | 12 | 0.050 | 40.33 | 2.02 |
|  | Calcium | 23.65 | 75 | 0.050 | 31.533 | 1.58 |
|  | Magnesium | 4.86 | 50 | 0.025 | 9.72 | 0.24 |
|  |  |  |  |  | WQI = ∑SI = 53.5 | |

Table S6 Calculation of WQI at Baluwatar (RO5)

| **S.N** | **Parameters** | **Observed values (C_i_)** | **Standard values (S_i_)** | **Relative weight (W_r_)** | **Quality rating (Q_i_)** | **Sub-indices (SI) values** |
| --- | --- | --- | --- | --- | --- | --- |
|  | PH | 7.5 | 7.5 | 0.100 | 100 | 10 |
|  | EC | 265 | 300 | 0.125 | 88.333 | 11.04 |
|  | TDS | 177 | 500 | 0.100 | 35.4 | 3.54 |
|  | DO | 5.4 | 6 | 0.125 | 106.977 | 13.37 |
|  | Turbidity | 2.70 | 5 | 0.100 | 54 | 5.40 |
|  | Chloride | 8 | 250 | 0.075 | 3.2 | 0.24 |
|  | TH | 84 | 500 | 0.050 | 16.8 | 0.84 |
|  | TA | 90.10 | 200 | 0.050 | 45.05 | 2.25 |
|  | Sulphate | 23 | 250 | 0.100 | 9.2 | 0.92 |
|  | Sodium | 7.53 | 200 | 0.050 | 3.765 | 0.19 |
|  | Potassium | 5.98 | 12 | 0.050 | 49.833 | 2.49 |
|  | Calcium | 26.05 | 75 | 0.050 | 34.733 | 1.74 |
|  | Magnesium | 4.62 | 50 | 0.025 | 9.24 | 0.23 |
|  |  |  |  |  | WQI = ∑SI = 52.1 | |

Table S7 Calculation of WQI at Setumari (RO6)

| **S.N** | **Parameters** | **Observed values (C_i_)** | **Standard values (S_i_)** | **Relative weight (W_r_)** | **Quality rating (Q_i_)** | **Sub-indices (SI) values** |
| --- | --- | --- | --- | --- | --- | --- |
|  | PH | 7.6 | 7.5 | 0.100 | 120 | 12 |
|  | EC | 285 | 300 | 0.125 | 95 | 11.87 |
|  | TDS | 189 | 500 | 0.100 | 37.8 | 3.78 |
|  | DO | 6.0 | 6 | 0.125 | 100 | 12.50 |
|  | Turbidity | 9.80 | 5 | 0.100 | 196 | 19.60 |
|  | Chloride | 1 | 250 | 0.075 | 0.4 | 0.03 |
|  | TH | 80 | 500 | 0.050 | 16 | 0.80 |
|  | TA | 103.70 | 200 | 0.050 | 51.85 | 2.59 |
|  | Sulphate | 44 | 250 | 0.100 | 17.6 | 1.76 |
|  | Sodium | 7.07 | 200 | 0.050 | 3.535 | 0.18 |
|  | Potassium | 5.26 | 12 | 0.050 | 43.833 | 2.19 |
|  | Calcium | 30.86 | 75 | 0.050 | 41.147 | 2.06 |
|  | Magnesium | 0.73 | 50 | 0.025 | 1.46 | 0.04 |
|  |  |  |  |  | WQI = ∑SI = 69.4 | |
